# Achieving Dissemination and Implementation Success in Dementia Care: A Consideration of Methodological Strategies

**DOI:** 10.1002/alz70858_096546

**Published:** 2025-12-24

**Authors:** Joseph Gaugler

**Affiliations:** ^1^ University of Minnesota, Minneapolis, MN, USA

## Abstract

**Background:**

With the passage of the National Alzheimer's Project Act (NAPA) in 2011 and the National Plan to Address Alzheimer's Disease in the U.S., federal funding support for dementia care science increased exponentially. The National Institute on Aging (NIA), in keeping with National Plan's goal of effectively treating and preventing Alzheimer's disease and Alzheimer's Disease Related Dementias (AD/ADRD) by 2025, created a series of research milestones to track success. One of these is research milestone 13.H: *Create research programs to evaluate novel and innovative dissemination and implementation methods to scale up promising practices in dementia care across settings and across the disease severity spectrum*. The objective of this presentation is to summarize scientific advancements in the dissemination and implementation of dementia care innovations.

**Method:**

This presentation uses narrative review and case study methods to assess the state‐of‐the‐science of dementia care implementation and dissemination and to identify recommendations to continue to propel the field forward so that the ultimate goal of ensuring people living with dementia and their carers can benefit from evidence‐based programs, services, and innovations are more readily achieved.

**Result:**

Although a range of efforts exist documenting dissemination and implementation efforts of dementia care interventions, a range of barriers have been identified in the literature, including staff training requirements, length, complexity, little alignment with operations and daily workflow, and lack of incentivization/reimbursement. Within the framework of the NIH Stage Model of Behavioral Intervention Development, several methodological strategies could be applied to dementia care intervention design, testing, and dissemination/implementation to expedite the translation of evidence into real‐world contexts.

**Conclusion:**

Designing dementia care interventions with the intent of implementation at the earliest stages of development is critical. Key methodological strategies to expedite the translational “pipeline” of dementia care science include the identification, measurement, and testing of mechanisms of action; use of process evaluations; application of advanced intervention design methodologies such as the Multiphase Optimization Strategy, Sequential Multiple Assignment Randomized Trials, and hybrid‐effectiveness studies; and incorporation of person‐centered outcome measures.

